# Tumor-associated macrophages in classical Hodgkin lymphoma: hormetic relationship to outcome

**DOI:** 10.1038/s41598-020-66010-z

**Published:** 2020-06-10

**Authors:** Laura Werner, Johannes H. Dreyer, David Hartmann, Mário Henrique M. Barros, Maike Büttner-Herold, Ulrike Grittner, Gerald Niedobitek

**Affiliations:** 1 Institute for Pathology, Unfallkrankenhaus Berlin, Berlin, Germany; 2 Department of Nephropathology, Institute of Pathology, Friedrich-Alexander-University Erlangen Nuremberg, Erlangen, Germany; 3 Institute of Biometry and Clinical Epidemiology, Charité - Universitätsmedizin Berlin, Berlin, Germany; 4 Berlin Institute of Health (BIH), Berlin, Germany; 5 Institute for Pathology, Sana Klinikum Lichtenberg, Berlin, Germany

**Keywords:** Hodgkin lymphoma, Cancer microenvironment

## Abstract

Commonly attributed to the prevalence of M2 macrophages, tumor-associated macrophages (TAM) are linked to poor outcome in Hodgkin lymphoma (HL). MYC is supposed to control the expression of M2-specific genes in macrophages, and deficiency in MYC-positive macrophages inhibits tumor growth in mouse models. To verify this hypothesis for HL, seventy-six samples were subjected to immunohistochemical double staining using CD68 or CD163 macrophage-specific antibodies and a reagent detecting MYC. For each cell population, labelled cells were grouped according to low, intermediate and high numbers and related to disease-free survival (DFS) and overall survival (OS). MYC+ cells accounted for 21% and 18% of CD68+ and CD163+ cells, respectively. Numbers of MYC− macrophages were significantly higher in EBV+ cases while no differences were observed for MYC+ macrophages between EBV+ and EBV− cases. Cases with highest numbers of macrophages usually showed worst DFS and OS. In most scenarios, intermediate numbers of macrophages were associated with better outcome than very low or very high numbers. Our observations are reminiscent of the “hormesis hypothesis” and suggest that a relative lack of TAM may allow HL growth while macrophages display an inhibitory effect with increasing numbers. Above a certain threshold, TAM may again support tumor growth.

## Introduction

Hodgkin lymphoma (HL) is classified into two major types: classical HL (cHL), accounting for 95% of all HL cases, and nodular lymphocyte predominant HL (NLPHL)^1,2^. Histologically, HL is characterized by a small number of neoplastic Hodgkin and Reed-Sternberg (HRS) cells embedded in an abundant inflammatory infiltrate of immune cells, including lymphocytes, macrophages, eosinophils, mast cells, plasma cells and stroma cells. These cells contribute to the microenvironment of the tumor^3,4^.

In general, macrophages play an important role in tumor microenvironments^5^. They are commonly defined according to the M1/M2-paradigm^6^ with M1-polarised macrophages considered as being cytotoxic, and M2-polarised macrophages as promoting wound healing, angiogenesis and defence against parasites. Consequently, M1 macrophages are generally considered to have anti-neoplastic effects while M2 macrophages are believed to support tumor growth. M1/M2-polarisation is influenced by the microenvironment^7,8^ and the ability of Th1/Th2 cytokines promoting opposing activities in macrophages^5,9–11^. For example, Th1 cells release interferon-γ, which activates the transcription factor STAT1^12^. STAT1 is responsible for the expression of various cytokines such as interleukin(IL)-12 as well as the expression of nitrogen-synthetase^13,14^. This activation of M1 macrophages is also called classical activation. “Alternative activation” of M2-polarised macrophages is supported by Th2 through release of IL-4, IL-10 and IL-13^14,15^ which activate transcription factors, such as CMAF, thus stimulating expression of IL-10 and arginase^16,17^.

Several studies have demonstrated an association of predominantly M2-polarised tumor-associated macrophages (TAM) in different types of tumors with poor prognosis^18–25^. The M1/M2-paradigm has been confirmed repeatedly but the spectrum of macrophage differentiation is still incompletely understood. A newly identified population of MYC-positive macrophages has recently been found. In macrophages, MYC supports the expression of M2-specific genes like ALOX15, MRC1, SCARB1 and, thus, M2-polarisation^26^.

MYC is known as a transcription factor, which promotes cell growth and differentiation. MYC also controls the expression of the tumor-promoting factors matrix metallopeptidase 9 (MMP9), vascular endothelial growth factor (VEGF), transforming growth factor (TGF-ß) and hypoxia-inducible factor-1 (HIF-1α)^26^. The deletion of the MYC gene in macrophages results in a reduced expression of these factors, a defective tumor angiogenesis and a reduced tumor growth in mouse melanoma and fibrosarcoma models^27^. Thus, the detection of MYC-positive macrophages is associated with increased tumor growth.

Epstein-Barr virus (EBV) infection is associated with the pathogenesis of cHL^28–30^. EBV status of HRS cells effects local immune response and macrophage differentiation. Barros *et al*.^31^ described a characteristic Th1 profile in EBV+ pediatric cHL with dominant M1-polarised microenvironment. By contrast, EBV− pediatric cHL display a predominant Th2 immune response, which is associated with a higher number of M2 macrophages^32^. In pediatric cHL, EBV absence is associated with poor disease outcome, while in adult cHL, conflicting evidence is shown and EBV+ cases are discussed to link to adverse prognosis^33–35^.

Here, we have examined a well characterised series of cHL cases for the presence of CD68+ and CD163+ macrophages with special emphasis on MYC+ macrophages and their impact on disease outcome in relation to EBV-status.

## Materials and Methods

### Tissue samples

Formalin-fixed paraffin-embedded (FFPE) tissue blocks from 84 patients diagnosed with HL at the Institute of Pathology, University of Erlangen (Germany) and collected between 1991 and 2007 were incorporated, including 53 cases of cHLns (cHL nodular sclerosis), 29 cases of cHLmc (cHL mixed cellularity), one case of cHLlr (cHL lymphocyte-rich) and one case of interfollicular cHL. The collective of 84 patients was partially part of a previous study^36^. Hodgkin’s lymphomas were classified according to the Ann Arbor classification. Fourteen cases corresponded to stage I, 36 cases to stage II, 17 cases to stage III, 11 cases to stage IV, in 6 cases the stage was unknown. All patients were treated according to the ongoing protocols of the German Hodgkin Lymphoma Study Group.

Fifty-three samples were from men and 31 from women. The age range at diagnosis was between 4 and 84 years, the average age 40 years. Eleven patients (13%) were 18 years old or younger at the time of diagnosis.

After staining the slides, eight cases had to be excluded because of tissue loss. Thus, 76 cases could be used for analysis (Table 1), including 51 cases of cHLns, 23 cases of cHLmc, 1 case of cHLlr and 1 case of interfollicular cHL (Table 1).

**Table 1:** Overview of the clinicopathological data (cHLns: nodular sclerosis classical Hodgkin lymphoma, cHLmc: mixed cellularity classical Hodgkin lymphoma, cHLlr: lymphocyte-rich classical Hodgkin lymphoma).

Characteristic	n (%)
cHL cases	76 (100)
cHLns	51 (67.1)
cHLmc	23 (30.3)
cHLlr	1 (1.3)
cHL interfollicular	1 (1.3)
**Gender**	
Male	46 (61)
Female	30 (39)
**EBV status**	
negative	50 (66)
positive	26 (34)
**Ann-Arbor**	
Stage I	13 (17)
Stage II	32 (42)
Stage III	14 (18)
Stage IV	11 (15)
Stage not known	6 (8)
Age at diagnosis in years (mean (SD))	40 (21)
Follow up in years (mean (SD), median [IQR])	7.6 (5.0), 6.7 [3.5–11.5]
Relapse	14 events
Death (any cause)	18 events
Tumor-caused death	10 events

All tissue samples had been submitted for diagnostic purposes and were examined for the purpose of this study only after completion of diagnosis. No material was taken for the sole purpose of this study.

### Double staining immunohistochemistry

Tissue microarrays were produced for all 84 HL cases with a core diameter of 1.5 mm. Two cores were processed per case.

Paraffin sections were prepared at a thickness of 1 μm, dewaxed and subjected to antigen retrieval in a Leica BOND III System (Leica, Berlin, Germany) by heating with HIER (heat induced epitope retrieval) EDTA buffer (Bond Epitope Retrieval Solution 2, pH 9.0, Leica BOND III System). Next, double staining immunohistochemistry was carried out using antibodies directed against macrophage-specific antigens (CD68, CD163) and against the proto-oncogene-encoded MYC protein using established protocols^37^.

Briefly, tissue samples were incubated with an appropriately diluted MYC antibody (1:50, monoclonal, rabbit, clone EP121; Biocare Medical, Pacheco, California, USA) as a primary antibody for 30 minutes at room temperature, followed by washing (Bond Wash Solution Leica BOND III System) and detection of immobilised antibodies using the horseradish peroxidase polymer reagent (Bond Polymer Refine Detection Leica BOND III System) and diaminobenzidine (DAB) as chromogen.

After washing (five minutes at room temperature) with Wash Buffer (Zytomed Systems, Berlin, Germany), anti-CD68 (1:800, clone PG-M1; Agilent Dako, Santa Clara, California, USA) or anti-CD163 (1:400, clone 10D6; Leica, Newcastle Upon Tyne, UK) antibodies were added and the slides incubated for 30 minutes at room temperature. Bound antibodies were detected by means of the Alkaline Phosphatase Polymer Kit (Zytomed Systems, Berlin, Germany) and Blue Alkaline Phosphatase (Vector Laboratories, California, USA) as a chromogen, following the protocols described by Barros *et al*.^37^. The slides were not counterstained.

Each sample was evaluated for total numbers of CD68+ and CD163+ macrophages, for CD68+/MYC+ and CD163+/MYC+ as well as for CD68+/MYC− and CD163+/MYC− cells.

### EBV detection

Samples had been tested previously for EBV infection by EBER-specific *in situ* hybridization (ISH) and/or by immunohistochemistry (IHC) targeting the EBV latent membrane protein 1 (LMP1) in the Institute of Pathology, University of Erlangen, Germany.

### Computer assisted microscopical analysis

Processed samples were visually examined using an Olympus microscope BX53 at 20x objective magnification and photographed at a scale of 1:200 (Olympus DP26). Two representative areas were photographed per case (one for each core). Criteria of image selection were areas around tumor cells with an increased occurrence of macrophages. Areas of sclerosis were excluded.

Labelled cells were counted per mm^2^, applying the image analysis software ImageJ (Java, Wisconsin, USA). For statistical analysis, the mean of the two cores per case was used.

### Statistical analysis

The statistical software SPSS (IBM SPSS STATISTICS 24) was used for all analyses, including statistical evaluation and graphical presentation, except for confidence interval calculation for event estimates which was done by R and R Studio^38^, package “survival”^39,40^. The measure of discrepancy between two related samples was evaluated by Wilcoxon signed-rank test. To check for group differences in continuous variables, Mann-Whitney-U test was used. Estimates for disease-free (DFS) and overall survival (OS) were calculated according to Kaplan-Meier. OS was defined as the time interval between initial diagnosis and death, DFS as the time interval between initial diagnosis and relapse. OS or DFS times between patient groups were compared with the log-rank test.

Cox regression analysis was used to additionally adjust for EBV-status. A two-sided significance level of α = 0.05 was used. No adjustment for multiple testing was applied in this exploratory study.

Survival ROC (Receiver operating characteristic) curves for CD163+, CD163+/MYC−, CD163+/MYC+ at time point 2 years after diagnosis were calculated for relapse and death of any cause. For promising cut off values for relapse, sensitivity, specificity and positive and negative predictive values were calculated. Survival ROC analyses was done using R package timeROC^41,42^.

### Ethics declarations

No humans were directly involved in the study. All cases have been included in previous studies^36,43^ and were available to us only as tissue microarrays in an anonymous fashion. All tissue samples were obtained for diagnostic purposes. The use of archival FFPE tissue blocks and of clinical data has been approved by the Ethics Committee of the Friedrich-Alexander-University of Erlangen-Nuremberg^36,43^.

## Results

### Macrophage subsets

**Figure 1:**
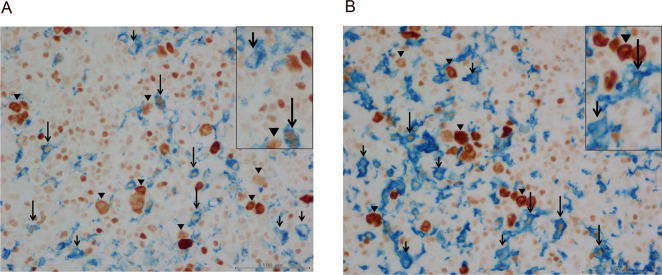
Examples of cHL biopsies stained by immunohistochemical double staining. Blue cytoplasmic/membranous staining indicates CD68 (**A**) or CD163 (**B**) expression, brown nuclear staining expression of transcription factor MYC (Fig. 1). Long arrows indicate MYC+ macrophages with blue cyctoplasmic/membraneous staining for CD68 (**A**) or CD163 (**B**) and usually moderate nuclear brown staining for MYC. Short arrows indicate macrophages lacking co-expression of MYC. In addition, there are large Hodgkin and Reed-Sternberg cells showing strong brown nuclear staining indicating MYC expression in the absence of macrophage-specific antigens (arrowheads). Finally, there are large numbers of small to medium-sized moderately MYC+ cells lacking expression macrophage antigens probably representing background lymphocytes.

76 HL samples were investigated by immunohistochemical double staining for expression of MYC protein and of macrophage-specific antigens CD68 or CD163 (Fig. 1). Numbers of CD68+ and CD163+ cells, double-positive (CD68+/MYC+, CD163+/MYC+) and single-positive (CD68+/MYC−, CD163+/MYC−) cells were analysed for each HL case. Results are summarised in Table 2 and Supplementary Fig. S1.

**Table 2:** Numbers of macrophages in cHL per mm^2^ (n = 76) in total and according to EBV status (n_EBV−_ = 50, n_EBV+_ = 26).

	CD68+/mm^2^		CD68+/MYC−/mm^2^		CD68+/MYC+/mm^2^		CD163+/mm^2^		CD163+/MYC−/mm^2^		CD163+/MYC+/mm^2^	
Mean (SD)	859 (255)		669 (203)		190 (106)		1175 (770)		940 (625)		234 (193)	
Median [IQR]	837 [686–995]		624 [537–779]		178 [113–248]		903 [602–1657]		754 [476–1257]		166 [106–316]	
	**EBV−**	**EBV+**	**EBV−**	**EBV+**	**EBV−**	**EBV+**	**EBV−**	**EBV+**	**EBV−**	**EBV+**	**EBV−**	**EBV+**
Mean (SD)	813 (252)	947 (240)	619 (184)	766 (205)	194 (107)	182 (104)	985 (630)	1538 (889)	773 (486)	1263 (736)	213 (174)	275 (224)
Median [IQR]	747 [623–949]	937 [769–1096]	596 [515–691]	740 [578–936]	179 [113–270]	178 [109–228]	840 [472–1456]	1246 [852–2172]	699 [389–974]	923 [700–1931]	152 [58–343]	216 [146–304]
p	0.012		0.002		0.65		0.004		0.002		0.158	

SD: standard deviation, IQR: interquartile range.

p (Mann-Whitney-U-Test).

Overall, higher numbers of CD163+ cells were detected compared to CD68+ cells (median 903 CD163+ macrophages/mm^2^ vs. median 837 CD68+ macrophages/mm^2^; p < 0.001 Wilcoxon signed-rank test; Table 2).

Median numbers of CD68+/MYC+ and CD163+/MYC+ macrophages were 178 and 166 cells/mm^2^, and 624 and 754 cells/mm^2^ for CD68+/MYC− and CD163+/MYC− macrophages (Table 2). Therefore, MYC+ cells accounted for on average 21% of CD68+ and 18% of CD163+ macrophages.

### EBV infection status and number of macrophages

EBV+ cases contained higher numbers of CD68+ and CD163+ macrophages/mm^2^ than EBV− cases (EBV+: median 937 CD68+ macrophages/mm^2^ and 1246 CD163+ macrophages/mm^2^, p = 0.012; EBV−: median 747 CD68+ macrophages/mm^2^ and 840 CD163+ macrophages/mm^2^; Table 2). On average, 20% more CD68+ and 33% more CD163+ macrophages were detected in EBV+ cases than in EBV− cases.

Higher numbers of CD68+/CMYC− and CD163+/CMYC− macrophages were observed in EBV+ cases than in EBV− cases (EBV+: median 740 CD68+/MYC− macrophages/mm^2^ and 923 CD163+/CMYC− macrophages/mm^2^; EBV−: median 596 CD68+/MYC− macrophages/mm^2^ and 699 CD163+/MYC− macrophages/mm^2^; p_CD68+/CMYC−_ = 0.002, p_CD163+/CMYC−_ = 0.002; Table 2). By contrast, no statistically significant differences between EBV+ and EBV− cases was observed regarding the numbers of MYC+ cells (Table 2).

### Macrophage numbers and survival analysis

To investigate the association of numbers of macrophages and outcome, cases were subdivided into three classes (tertiles) with low, intermediate and high numbers of cells for the total numbers of CD68+ and CD163+ macrophages, the numbers of MYC+ macrophages and the numbers of MYC− macrophages. The definition of classes is given in Supplementary Table S1.

**Figure 2:**
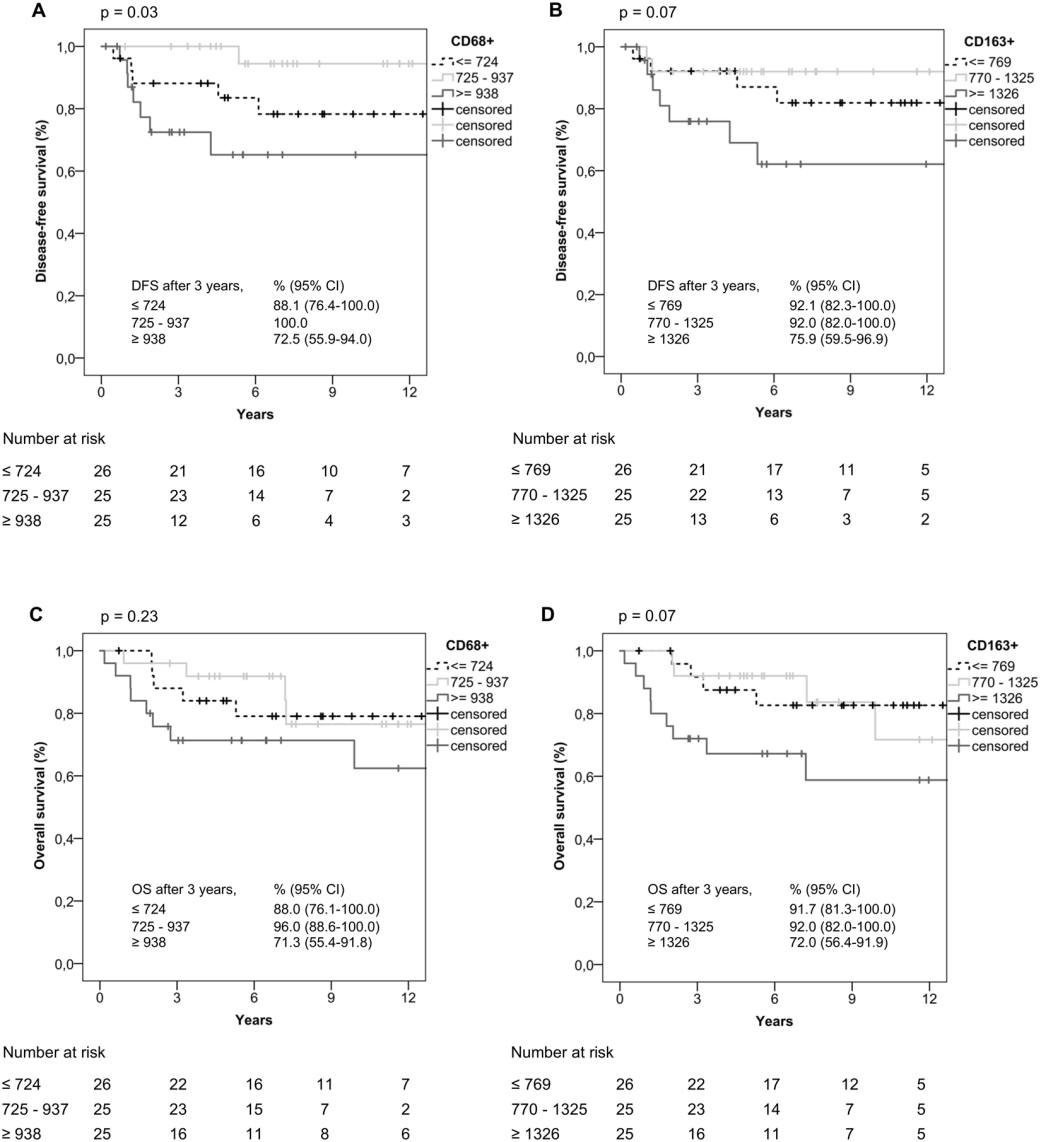
Kaplan-Meier curves in relation to number of macrophages (DFS for (**A**) CD68+ macrophages/mm^2^, (**B**) CD163+ macrophages/mm^2^, and OS for (**C**) CD68+ macrophages/mm^2^, (**D**) CD163+ macrophages/mm^2^; n = 76). Kaplan-Meier curves were done using IBM SPSS STATISTICS 24.

For total CD68+ and CD163+ cells, there were substantial differences in DFS with regards to tertiles of number of macrophages (CD68+: p = 0.03, CD163+: p = 0.07). Cases with highest numbers of macrophages (class 3) consistently displayed shortest DFS (Fig. 2A,B). Similarly, class 3 cases were characterized by shorter OS than class 1 and 2 cases (CD68+: p = 0.23, CD163+: p = 0.07; Fig. 2C,D). Unexpectedly, however, intermediate numbers of macrophages (class 2) were associated best DFS for both CD68+ and CD163+ cells whereas no substantial differences between class 1 and class 2 cases was observed in OS (Fig. 2). Overall, results of survival analyses were comparable for CD68+ and CD163+ groups.

**Figure 3:**
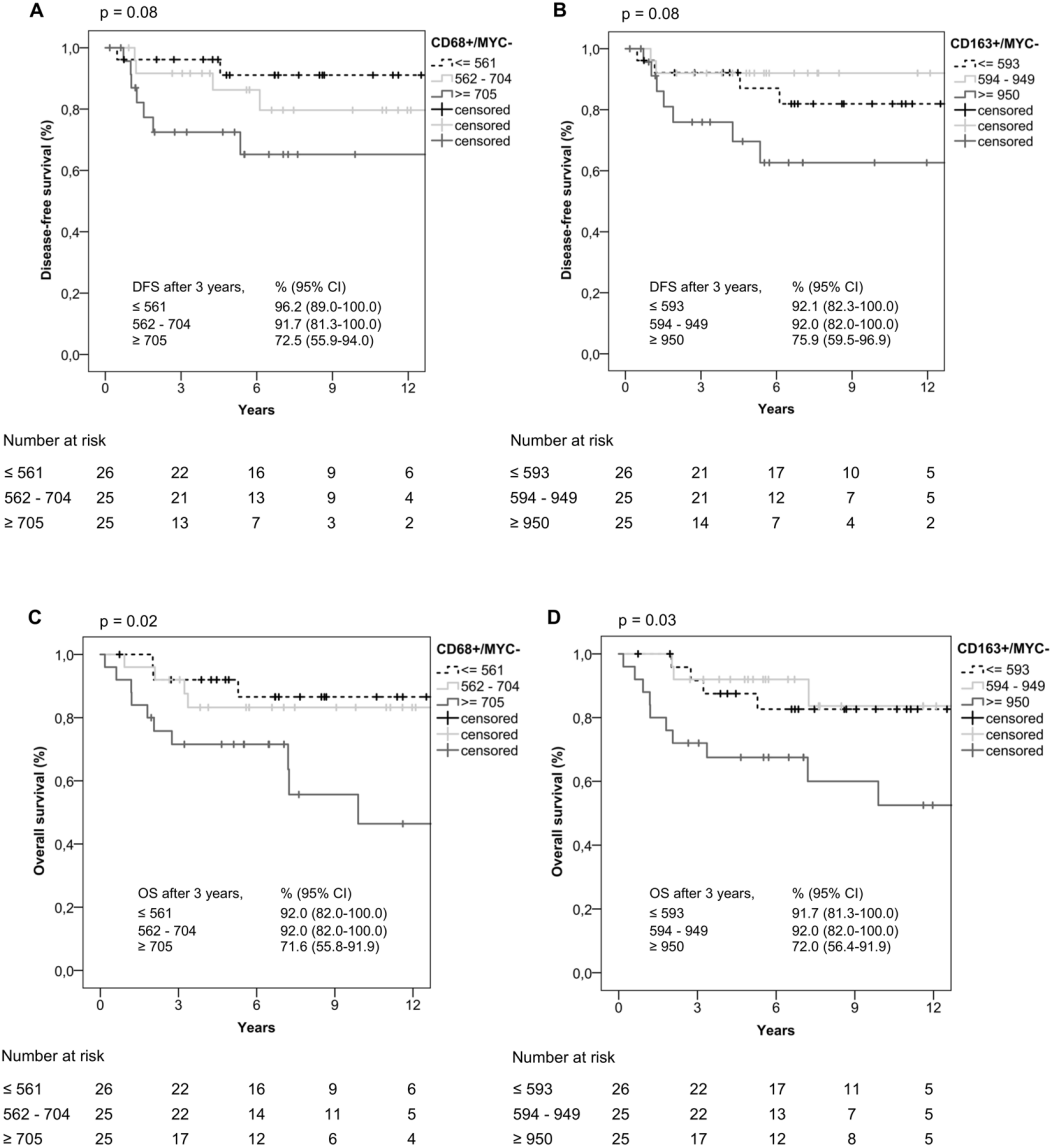
Kaplan-Meier curves in relation to MYC− macrophage distributions (**A**) CD68+/MYC− macrophages/mm^2^, (**B**) CD163+/MYC− macrophages/mm^2^ associated with DFS, (**C**) CD68+/MYC− macrophages/mm^2^, (**D**) CD163+/MYC− macrophages/mm^2^ associated with OS; n = 76). Kaplan-Meier curves were done using IBM SPSS STATISTICS 24.

Subgroups of MYC− and MYC+ macrophages were also subdivided into three classes (tertiles) as summarized in Supplementary Table S1. For MYC− macrophages, shorter DFS and OS were observed for class 3 cases (highest numbers of macrophages) than for class 1 and class 2 cases (Fig. 3A–D).

**Figure 4:**
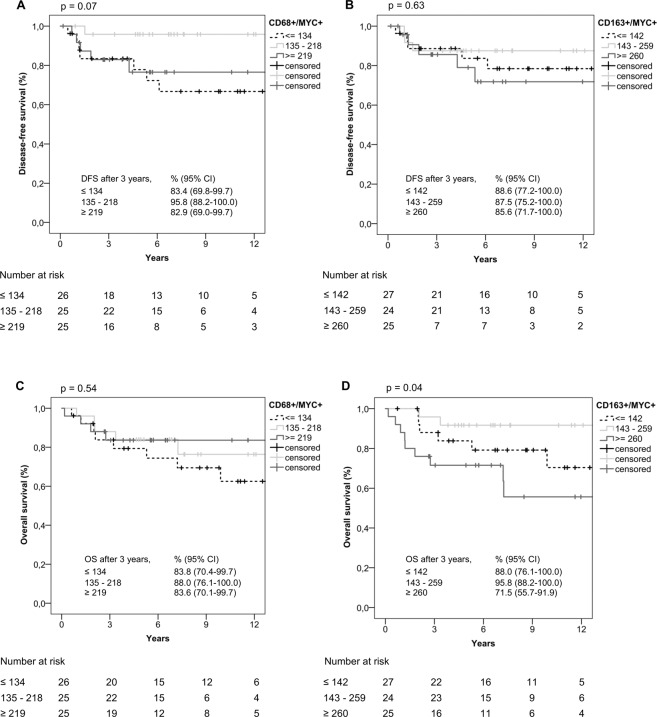
Kaplan-Meier curves in relation to MYC+ macrophage distributions (**A**) CD68+/MYC+ macrophages/mm^2^, (**B**) CD163+/MYC+ macrophages/mm^2^ associated with DFS, (**C**) CD68+/MYC+ macrophages/mm^2^, (**D**) CD163+/MYC+ macrophages/mm^2^ associated with OS; n = 76). Kaplan-Meier curves were done using IBM SPSS STATISTICS 24.

For MYC+ macrophages (Fig. 4A–D), high numbers of CD163+/MYC+ cells (class 3) were associated with shortest OS (Fig. 4D). Interestingly, best DFS was seen for cases with intermediate numbers of CD68+/MYC+ macrophages (class 2) and best OS occurred in class 2 cases with intermediate numbers of CD163+/MYC+ macrophages (Fig. 4A,D).

### Results ROC analysis

Using ROC analysis we analysed the association between CD163+, CD163+/MYC−, CD163+/MYC+ cells and DFS and OS (Supplementary Fig. S2 and Table S2). Area under the curve (AUC) were smaller than 0.5 for overall survival, however, for DSF AUC were between 0.62 and 0.75 (Supplementary Fig. S2). We also tried to find a cut off for DSF and calculated sensitivity, specificity and predictive values for promising cut offs. Sensitivity was 71%, specificity between 72% and 88%. Positive predictive values were low, between 20% and 39%, negative predictive values were between 85% and 89% (Supplementary Table S2). However, all these results should be interpreted with caution, since sample size is low.

### EBV infection status and survival

Overall, EBV infection of HRS cells was detected in 30 of 84 cases (36%).

In the EBV+ group, shorter DFS was observed compared to EBV− patients (Supplementary Fig. S3A). However, EBV status was not substantially associated with OS (Supplementary Fig. S3B).

Exploratory analyses stratified by EBV status suggested, that the association of macrophage numbers with DFS and OS was more pronounced in EBV+ cases than in EBV− cases (Kaplan Meier analyses, data not shown). In EBV+ cases, both highest total numbers of macrophages and of MYC− and MYC+ macrophages (class 3) were associated with shortest survival while cases with intermediate numbers of macrophages displayed best outcome in most scenarios although these effects were not statistically significant (not shown).

In Cox regression models including all cases (EBV+ and EBV−) it could be demonstrated that after adjustment for EBV status highest numbers of macrophages (class 3) were associated with shortest survival (Table 3, DFS and OS, class 3 compared to class 2) and cases with intermediate numbers of macrophages (class 2) displayed best DFS and OS in most scenarios (Table 3).

**Table 3:** Separate Cox regression analyses of DFS and OS adjusted for EBV infection status. Hazard ratios (HR) and 95% confidence intervals (CI).

	Cox regression analysis for DFS n = 75 cases/14 events			Cox regression analysis for OS n = 76 cases/18 events		
	HR	95% CI	p	HR	95% CI	p
**CD68**+						
cl. 1: ≤ 724	5.60	(0.65–48.08)	0.117	1.27	(0.34–4.76)	0.720
cl. 2: 725–937	1			1		
cl. 3: ≥ 938	10.23	(1.25–83.89)	**0.030**	2.26	(0.67–7.57)	0.188
**CD163**+						
cl. 1: ≤ 769	3.32	(0.54–20.32)	0.194	1.29	(0.29–5.72)	0.734
cl. 2: 770–1325	1			1		
cl. 3: ≥ 1326	5.48	(1.11–26.93)	**0.036**	2.86	(0.88–9.29)	0.081
**CD68**+**/MYC−**						
cl. 1: ≤ 561	0.46	(0.09–2.53)	0.374	0.73	(0.16–3.25)	0.675
cl. 2: 562–704	1			1		
cl. 3: ≥ 705	1.83	(0.51–6.60)	0.353	2.64	(0.77–9.06)	0.123
**CD163**+**/MYC−**						
cl. 1: ≤ 593	2.73	(0.47–15.76)	0.262	1.61	(0.34–7.60)	0.548
cl. 2: 594–949	1			1		
cl. 3: ≥ 950	4.90	(1.01–23.77)	**0.049**	3.92	(1.08–14.26)	**0.038**
**CD68**+**/MYC**+						
cl. 1: ≤ 134	9.55	(1.17–78.05)	**0.035**	1.72	(0.56–5.31)	0.344
cl. 2: 135–218	1			1		
cl. 3: ≥ 219	8.25	(0.95–71.89)	0.056	0.97	(0.26–3.65)	0.965
**CD163**+**/MYC**+						
cl. 1: ≤ 142	2.04	(0.47–8.86)	0.341	3.73	(0.73–19.02)	0.114
cl. 2: 143–259	1			1		
cl. 3: ≥ 260	2.20	(0.52–8.91)	0.282	5.86	(1.26–27.17)	**0.024**

## Discussion

Previous studies on the role of macrophages in the pathogenesis of cHL have generally concluded that high numbers of macrophages in the tumor microenvironment are associated with poor outcome in cHL patients^18,44–49^ although this has been disputed by others^50–52^. These studies usually employed CD68 antibodies for the immunohistochemical detection of macrophages. In other studies, this was combined with the detection of CD163, based on the assumption that CD163 is a marker of M2 polarisation^48,50,53^.

This assumption, however, has been challenged by the demonstration that expression of M1-specific transcription factors such as pSTAT1 can be detected in CD163+ macrophages^37,54^. Furthermore, we show here that CD163+ cell can outnumber CD68+ cells in cHL and that numbers of CD163+ cells are far greater in EBV+ than in EBV− cHL cases (p = 0.004, Table 2). In view of the prevalence of a Th1-predominant microenvironment in EBV+ cHL^32,55^, this observation provides further evidence to suggest that CD163+ cells cannot be considered as an M2 subgroup of total CD68+ macrophages. It is not clear why CD163+ cells can outnumber CD68+ cells. This would require an in depth phenotypic analysis of CD163+ cells. One possibility would be expression of CD163 in cells other than macrophages. Indeed, it has been reported that dendritic cells can express CD163^56^.

To assess the impact of total macrophages on outcome, we adopted a novel approach, assigning cases to three classes with low, intermediate, and high numbers of macrophages (Supplementary Table S1). Although limited by the relatively small case number as well as the small number of events, this analysis allowed a more subtle analysis of the effects of varying macrophages numbers on outcome in cHL than the two-tier system usually employed in previous studies^43,47–50^. Using this approach, we confirm previous papers showing that the numbers of TAM are critical for outcome in cHL and that highest numbers of TAM (class 3 in our study) are usually associated with worst outcome^18,47–49,53^. This was true for both CD68+ as well as for CD163+ cells. Studying an overlapping series of cases, Tudor *et al*. previously obtained similar results for CD163+ macrophages but not for CD68+ cells^43^. Unexpectedly, however, the three-tier approach adopted by us revealed that cases with intermediate numbers of macrophages (class 2) usually showed the best outcome, ahead of cases with smallest numbers of macrophages (Table 3). This was observed for total numbers of CD68+ and CD163+ macrophages, MYC+ and MYC− macrophages and applied to both DFS and OS, with the exception of CD68+/MYC− macrophages which showed best outcome in the group with lowest macrophage numbers (class 1). Use of a four-tiered system confirmed these observations (not shown). Thus, while confirming the negative prognostic impact of high numbers of macrophages in the cHL microenvironment, our data indicate that there is no linear dose-effect relationship and sheds new light onto the role of macrophages in cHL.

MYC has recently been identified as a key factor in the alternative activation of M2 macrophages^26^. Pello *et al*. showed a defective tumor angiogenesis and reduced tumor growth by deletion of the MYC gene in macrophages in mouse models of melanoma and fibrosarcoma^27^. There are no previous studies addressing the possible role of these MYC+ and MYC− macrophages in human tumor models. Here, we first demonstrate that numbers of MYC+ macrophages account for approximately 20% of all macrophages for both CD68+ and CD163+ cells. Interestingly, in EBV+ cHL cases, more MYC− macrophages were observed (CD68 p = 0.002, CD163 p = 0.002) while numbers of MYC+ macrophages were similar between EBV+ and EBV− cHL cases (Table 2). This is in line with the prevalent Th1 immune response pattern in EBV+ cHL^32^ and indeed suggests that MYC expression in human macrophages is related to M2 polarisation.

Based on the observations of Pello *et al*.^26,27^, we hypothesised that MYC+ macrophages would be associated with poor outcome relative to MYC− macrophages. In our analyses, CD163+/MYC− macrophages behaved similar to overall macrophages. Specifically, worst outcome was associated with highest macrophage numbers (class 3) and best outcome with intermediate numbers (class 2) for both DFS and OS. By contrast for CD68+/MYC− cells, best outcome was observed for cases with lowest numbers (class 1) for both endpoints. For MYC+ macrophages, Kaplan-Meier curves revealed no clear trend, but in Cox regression the pattern of best outcome with intermediate numbers (class 2) again emerged.

EBV is an established co-factor in the pathogenesis of cHL. However, it is uncertain if EBV contributes to outcome of cHL^33–35,57–59^, and this may also depend on patient age^31,33–35,59,60^. In our study, EBV+ cases displayed worse DFS and OS (p = 0.06 and p = 0.25, respectively) than EBV− cases. Confirming previous findings^43,47–50,60^, EBV+ cases were characterised by higher numbers of tumor-associated macrophages in our study. We therefore hypothesise that EBV-mediated cytokine and chemokine synthesis by HRS cells may contribute to the accumulation of macrophages in cHL microenvironment and through this mechanism to worse outcome.

Our observation that intermediate numbers of macrophages in cHL microenvironment may be associated with better outcome than very low and very high numbers is reminiscent of the “hormesis hypothesis” as proposed by Prehn for tumor infiltrating immune cells^61^. Prehn suggested that very small numbers of tumor-infiltrating immune cells may be insufficient to support tumor growth and only become supportive of tumor growth above a certain threshold. Further, tumor growth is inhibited by high doses of immune cells^61,62^.

Our data suggest an inverse situation in which small numbers of tumor-associated macrophages may have a moderate growth promoting effect on cHL (class 1 cases in our study) while with increasing numbers, macrophages display an inhibitory effect on tumor growth (class 2 cases). Finally, very high numbers (class 3 cases) may provide support for tumor growth. This conclusion is supported by the results of Pearce *et al*.^63^ who showed that tumor growth is inhibited by high doses of anti-tumor antibodies but stimulated by low antibody doses in animal models of colorectal cancer and Burkitt lymphoma^63^. Interestingly, in that study, the authors observed high numbers of tumor-associated macrophages in animals treated with the tumor supporting dose while in animals treated with a tumor inhibiting dose, numbers of tumor-associated macrophages were low^63^. Finally, the authors showed that the tumor promoting effect of antibodies was dependent on the presence of macrophages^63^.

It is likely, that these effects of tumor-associated macrophages on outcome in cHL may not only be related to total numbers of macrophages but may reflect underlying changes in macrophage polarization. In any case, the notion that macrophages in tumor microenvironments may have a hormetic rather than a linear relationship to outcome parameters merits further investigation and should be considered in studies of other tumor entities.

## Supplementary information

Supplementary information.
