# Supplementary material for: Tumor-associated macrophages in classical Hodgkin lymphoma: hormetic relationship to outcome
Source: Sci Rep. 2020 Jun 10;10:9410. doi: 10.1038/s41598-020-66010-z (PMC7287068; doi:10.1038/s41598-020-66010-z)

**Supplementary Information**

**for**

**Tumor-associated macrophages in classical Hodgkin lymphoma: hormetic relationship to outcome**

Laura Werner, Johannes H. Dreyer, David Hartmann, Mário Henrique M. Barros, Maike Büttner-Herold, Ulrike Grittner, Gerald Niedobitek

**Supplementary Data**

Supplementary Table S1. Numbers of macrophages subdivided into three classes with low, intermediate and high numbers of cells for the total numbers of CD68+ and CD163+ macrophages, MYC- and MYC+ macrophages/mm^2^.

|  | **CD68+** | **CD163+** | **CD68+/MYC-** | **CD163+/MYC-** | **CD68+/MYC+** | **CD163+/MYC+** |
| --- | --- | --- | --- | --- | --- | --- |
| **class 1** | ≤ 724 | ≤ 769 | ≤ 561 | ≤ 593 | ≤ 134 | ≤ 142 |
| **class 2** | 725 - 937 | 770 - 1325 | 562 - 704 | 594 - 949 | 135 - 218 | 143 - 259 |
| **class 3** | ≥ 938 | ≥ 1326 | ≥ 705 | ≥ 950 | ≥ 219 | ≥ 260 |

Supplementary Table S2: AUC for relapse /death at 2 years after diagnosis and Sensitivity, Specificity, positive and negative predictive values for relapse, for CD163+, CD163+/MYC-, CD163+/MYC+.

|  | **AUC relapse at 2 years** | **Cut off, Sensitivity (Sens), Specificity (Spec), positive predictive value (PPV), negative predictive value (NPV)** | **AUC death any cause at 2 years** |
| --- | --- | --- | --- |
| **CD163+** | 72.9 | Cut off: 1800 Sens: 71.4% Spec: 88.4% PPV: 38.5% NPV: 88.9% | 16.2 |
| **CD163+/MYC-** | 74.9 | Cut off: 1400 Sens: 71.4% Spec: 87.0% PPV: 35.7%  NPV: 88.7% | 21.0 |
| **CD163+/MYC+** | 61.7 | Cut off: 270 Sens: 71.4% Spec: 72.5% PPV: 20.8%  NPV: 84.6% | 2.7 |

Supplementary Figure S1. Dot plots with median comparing EBV positive with EBV negative cases with regard to number of macrophages.


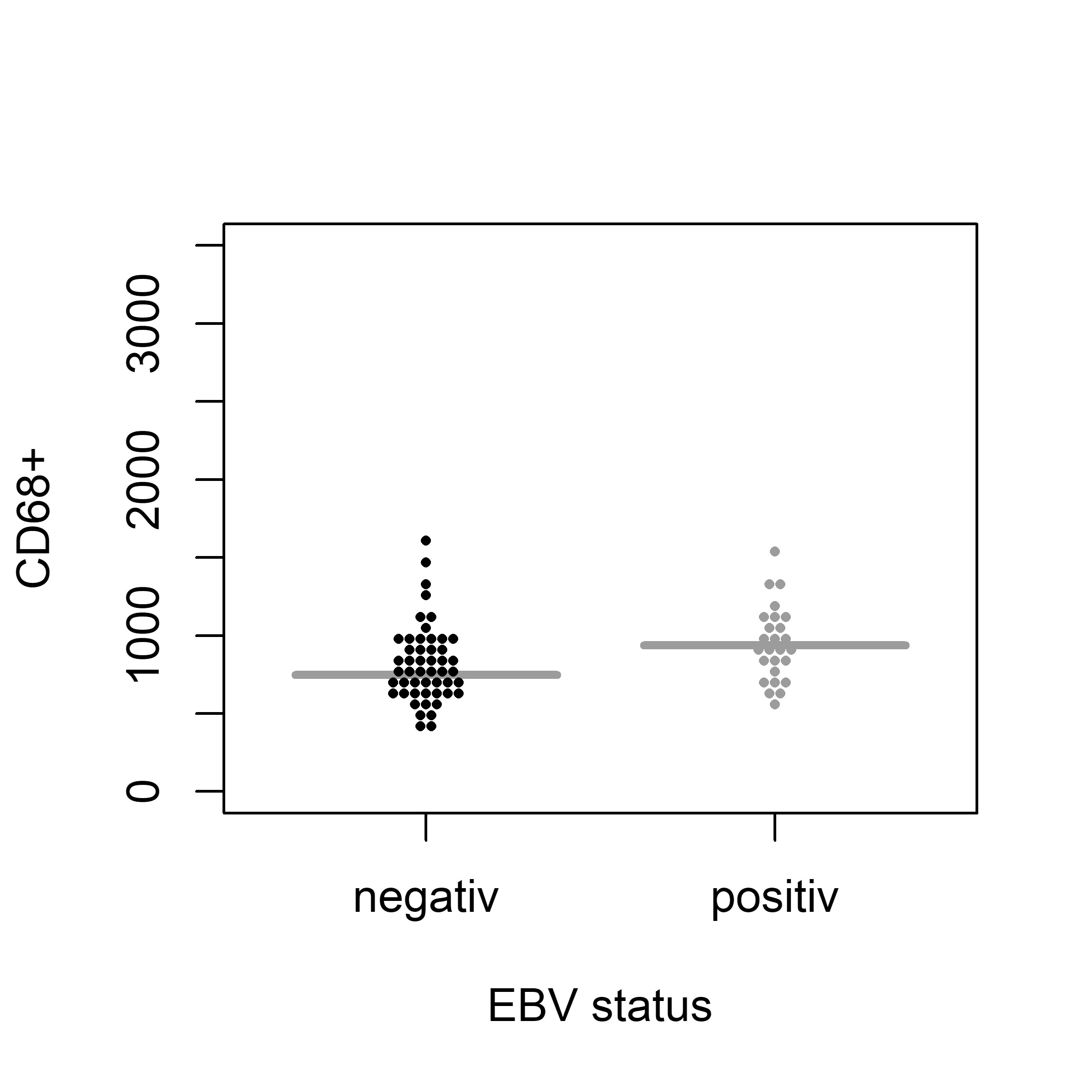

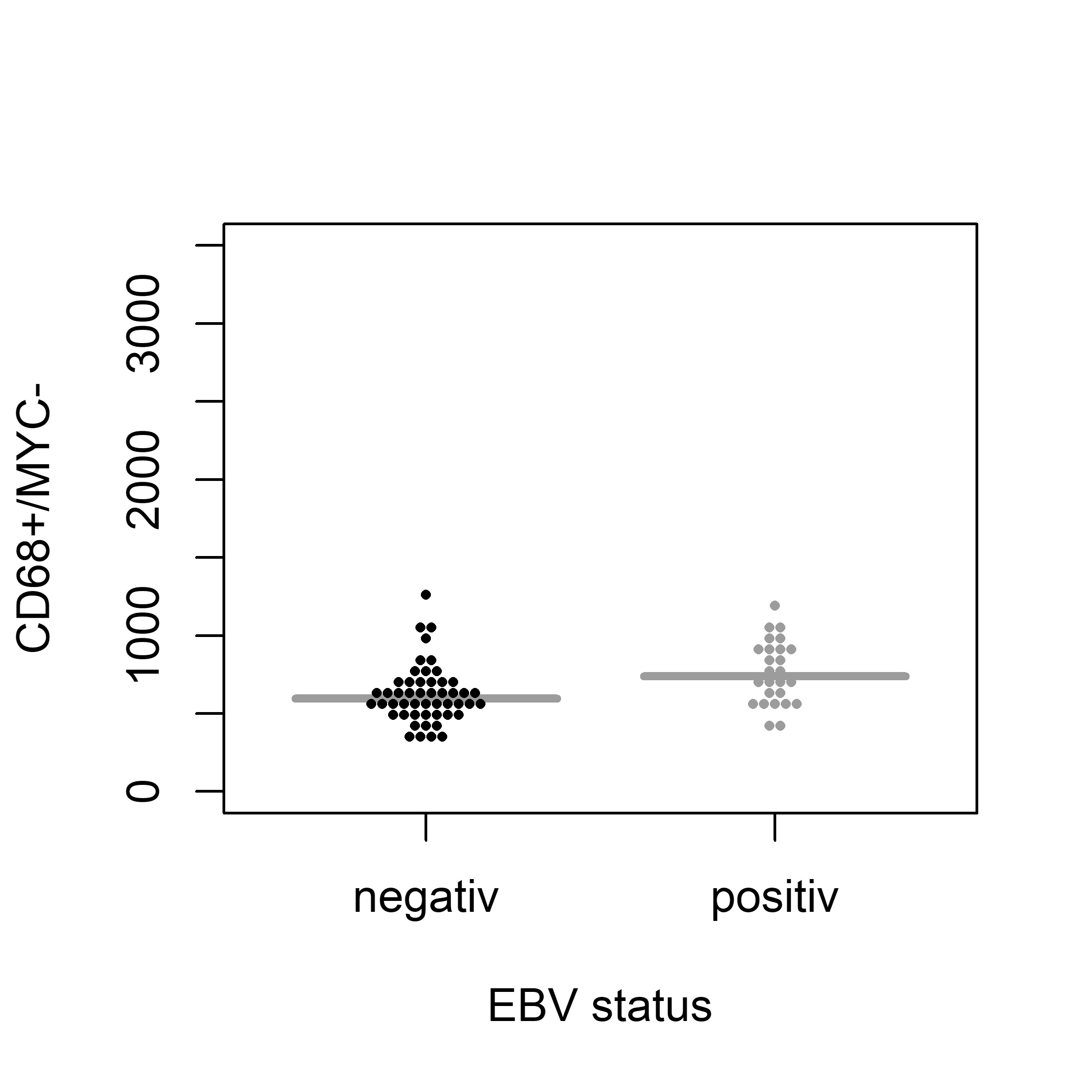

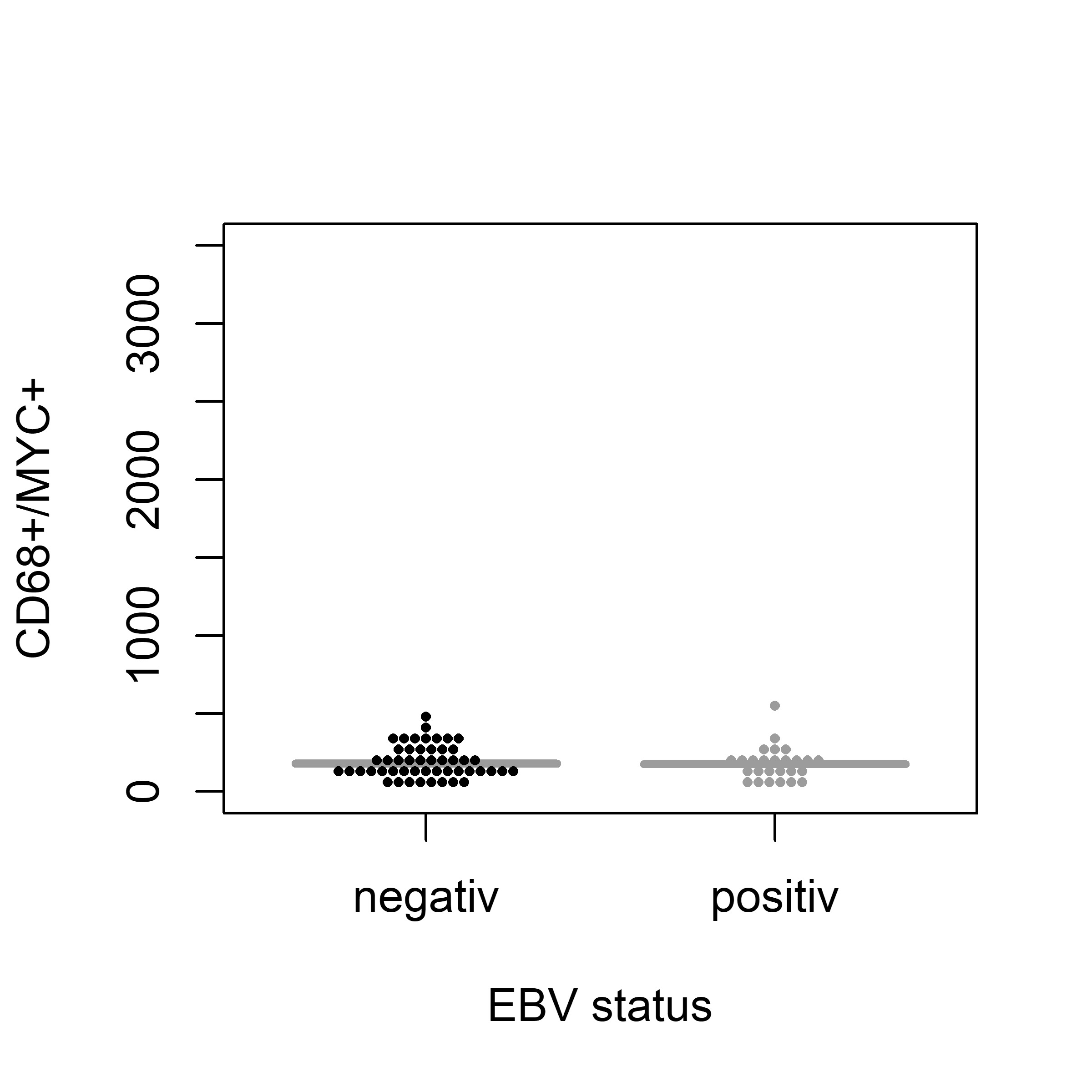


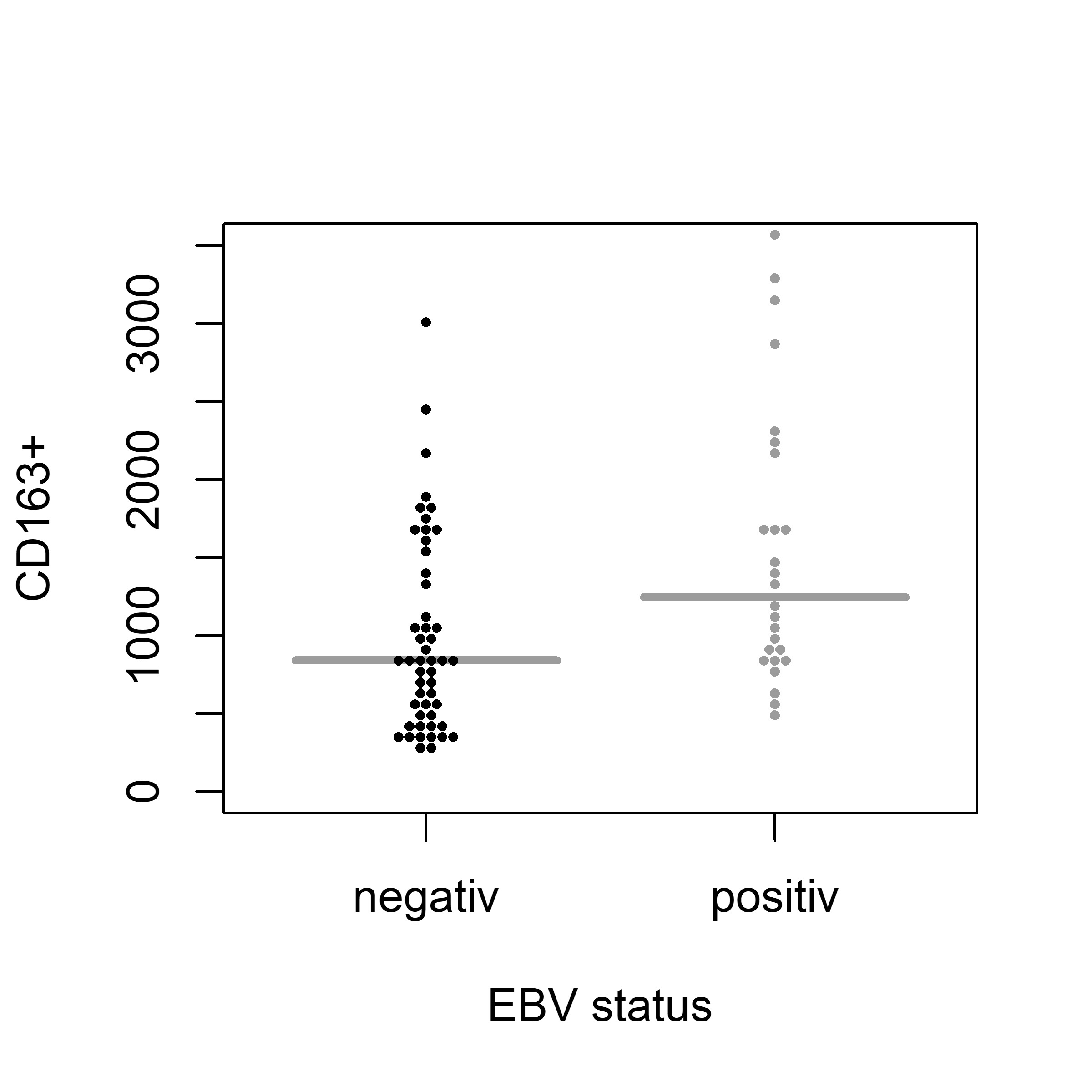

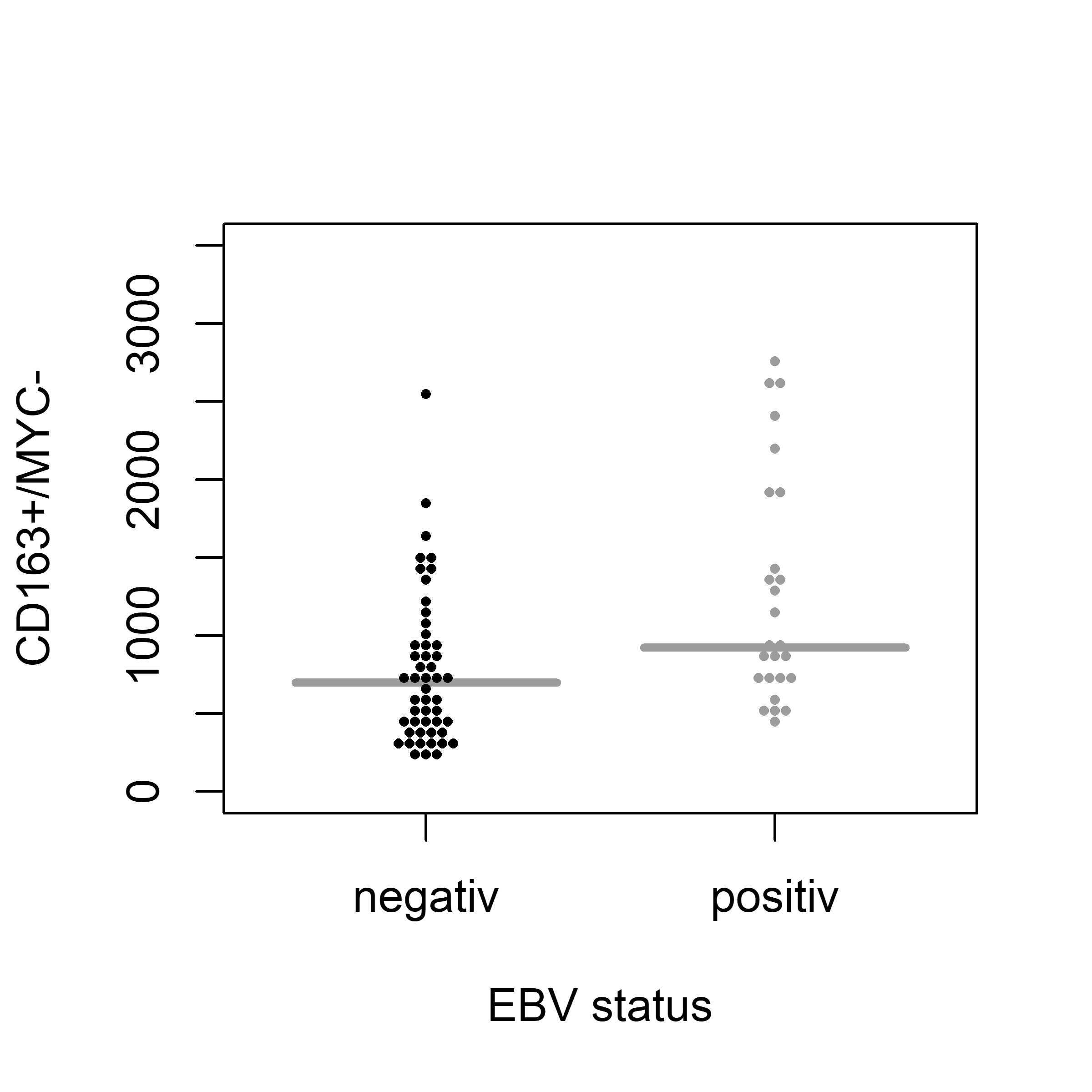

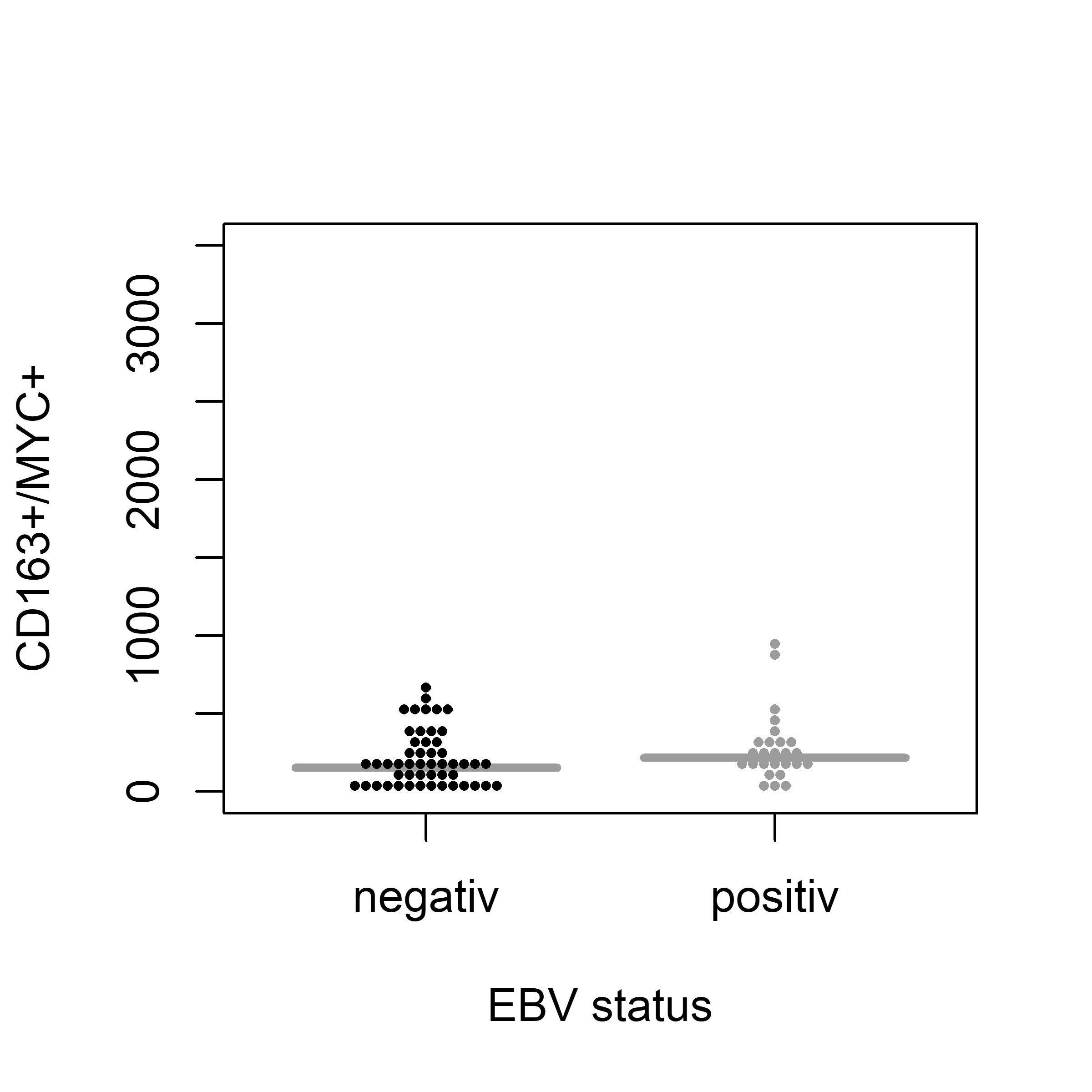


Supplementary Figure S2. Survival ROC Curves for relapse at 2 years after diagnosis for CD163+, CD163+/MYC-, CD163+/MYC+.

| **CD163+, Relapse at 2 years after diagnosis**  **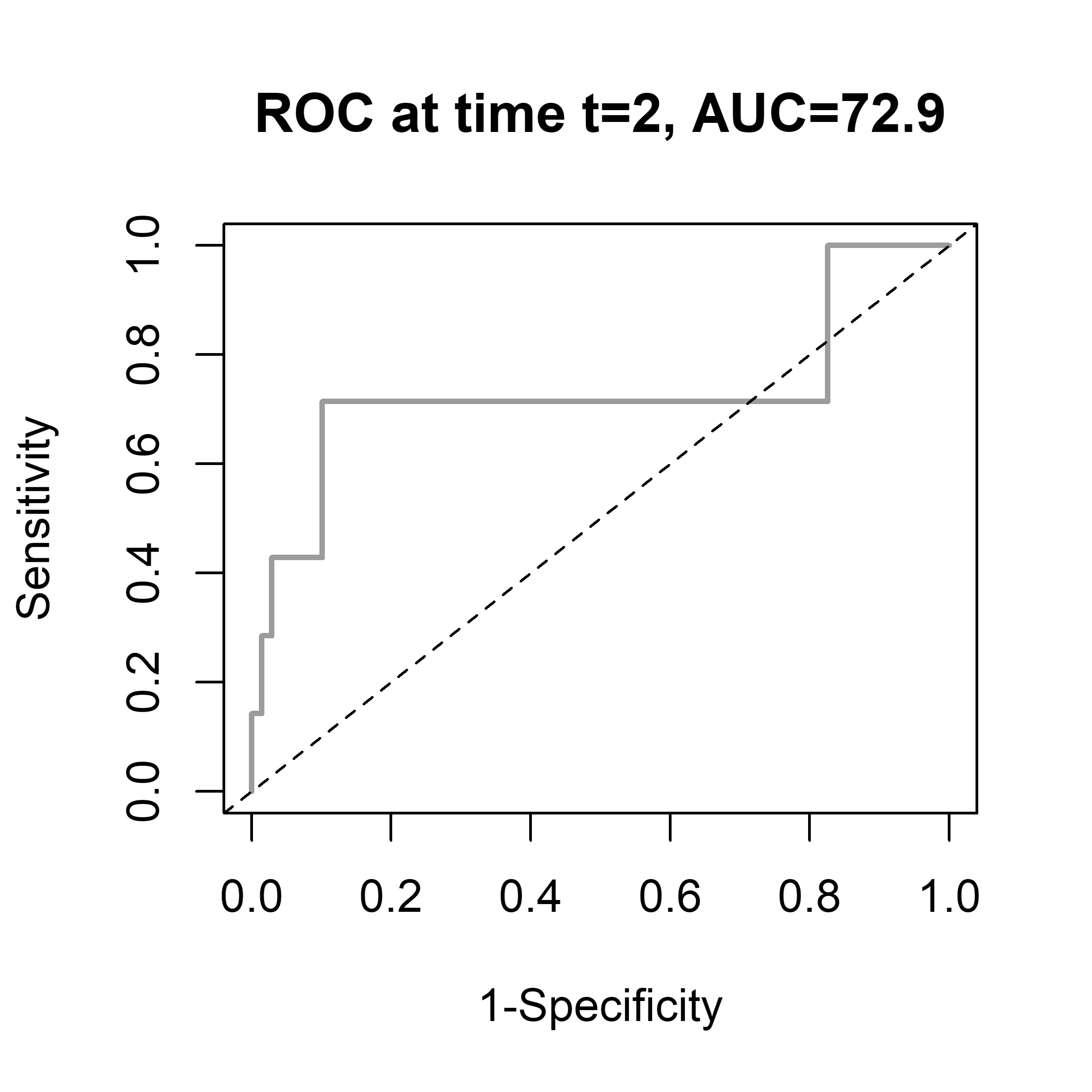** | **CD163+/MYC-, Relapse at 2 years after diagnosis**  **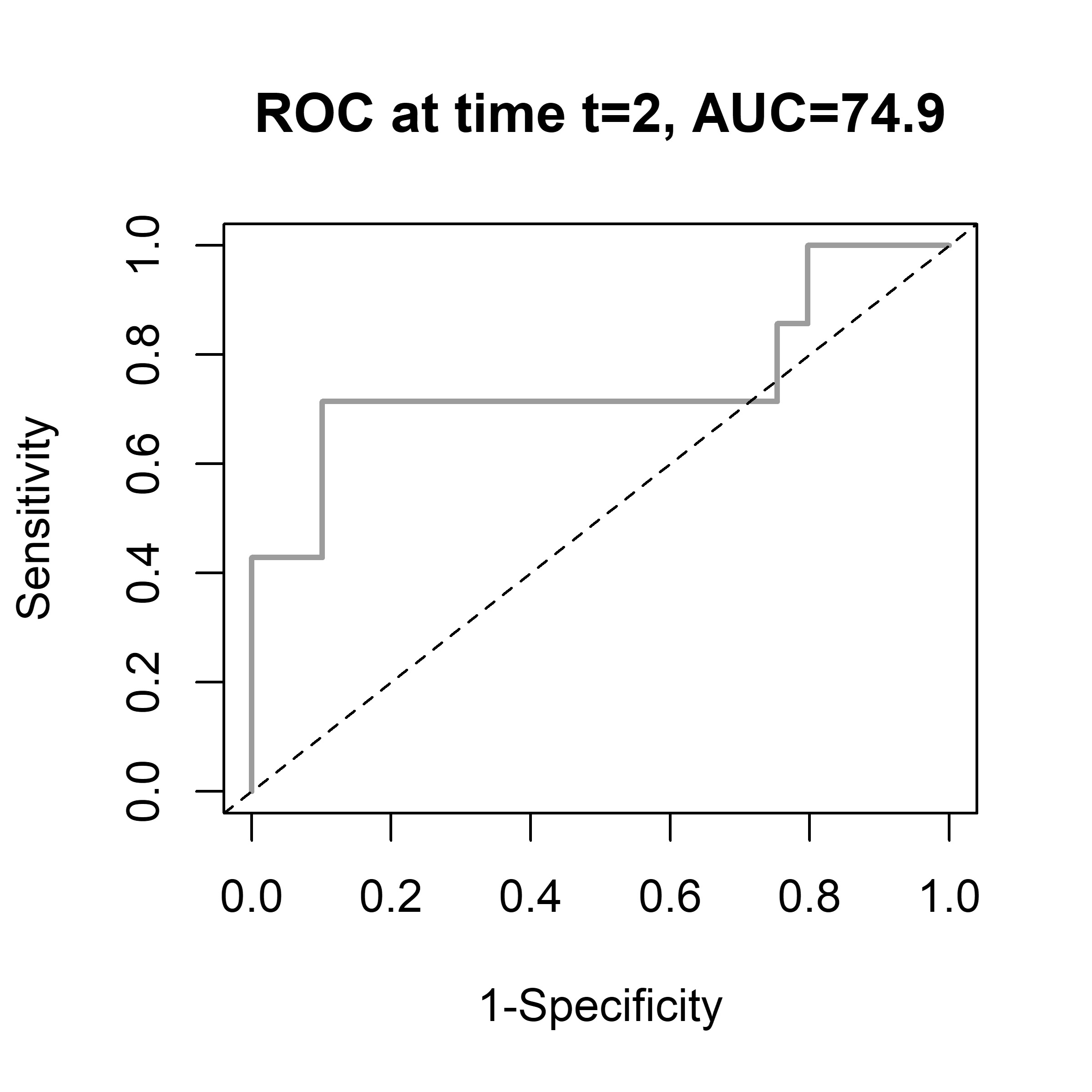** | **CD163+/MYC+, Relapse at 2 years after diagnosis**  **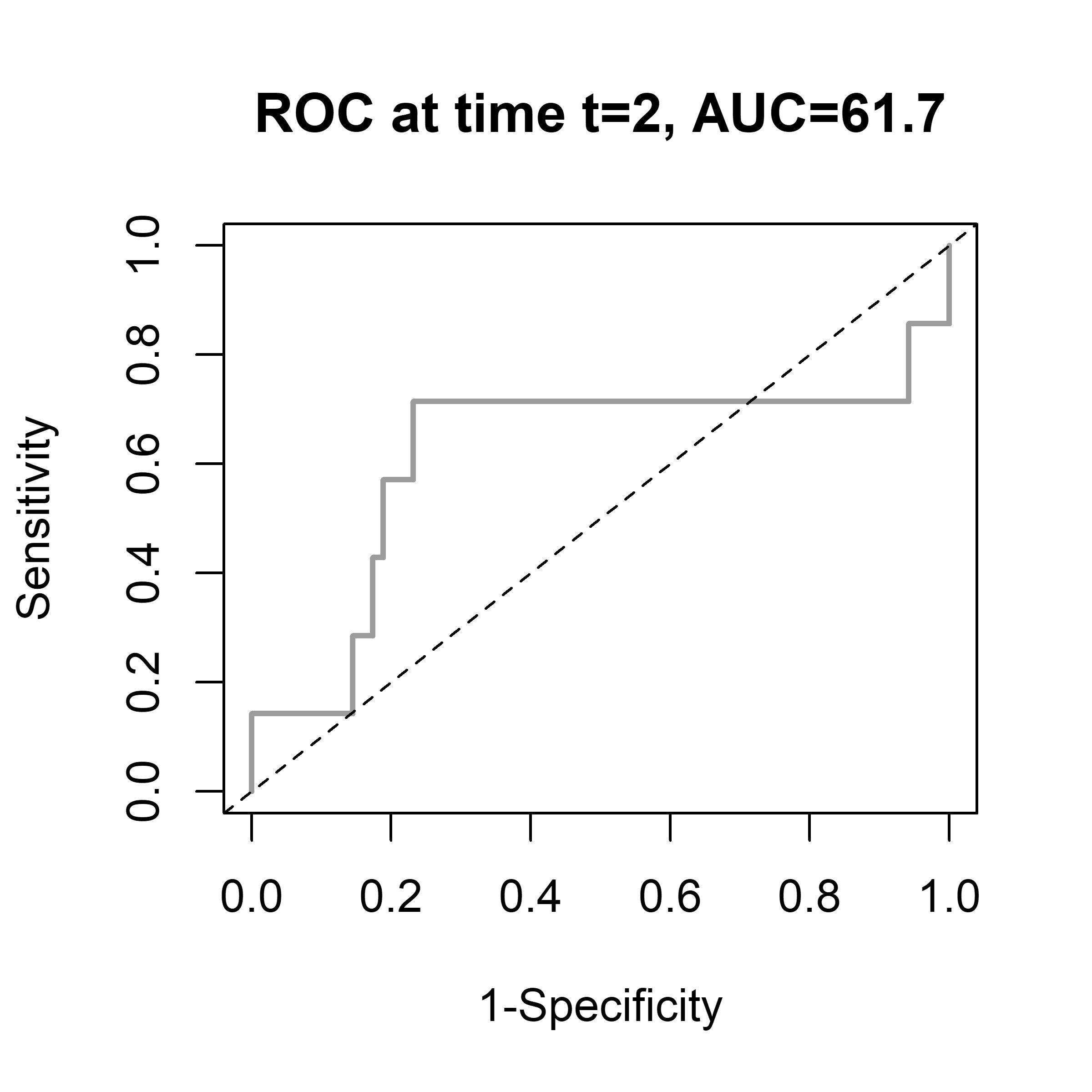** |
| --- | --- | --- |

Supplementary Figure S3. Kaplan-Meier curves of EBV status associated with the DFS (A) and OS (B) in cHL; n = 84.


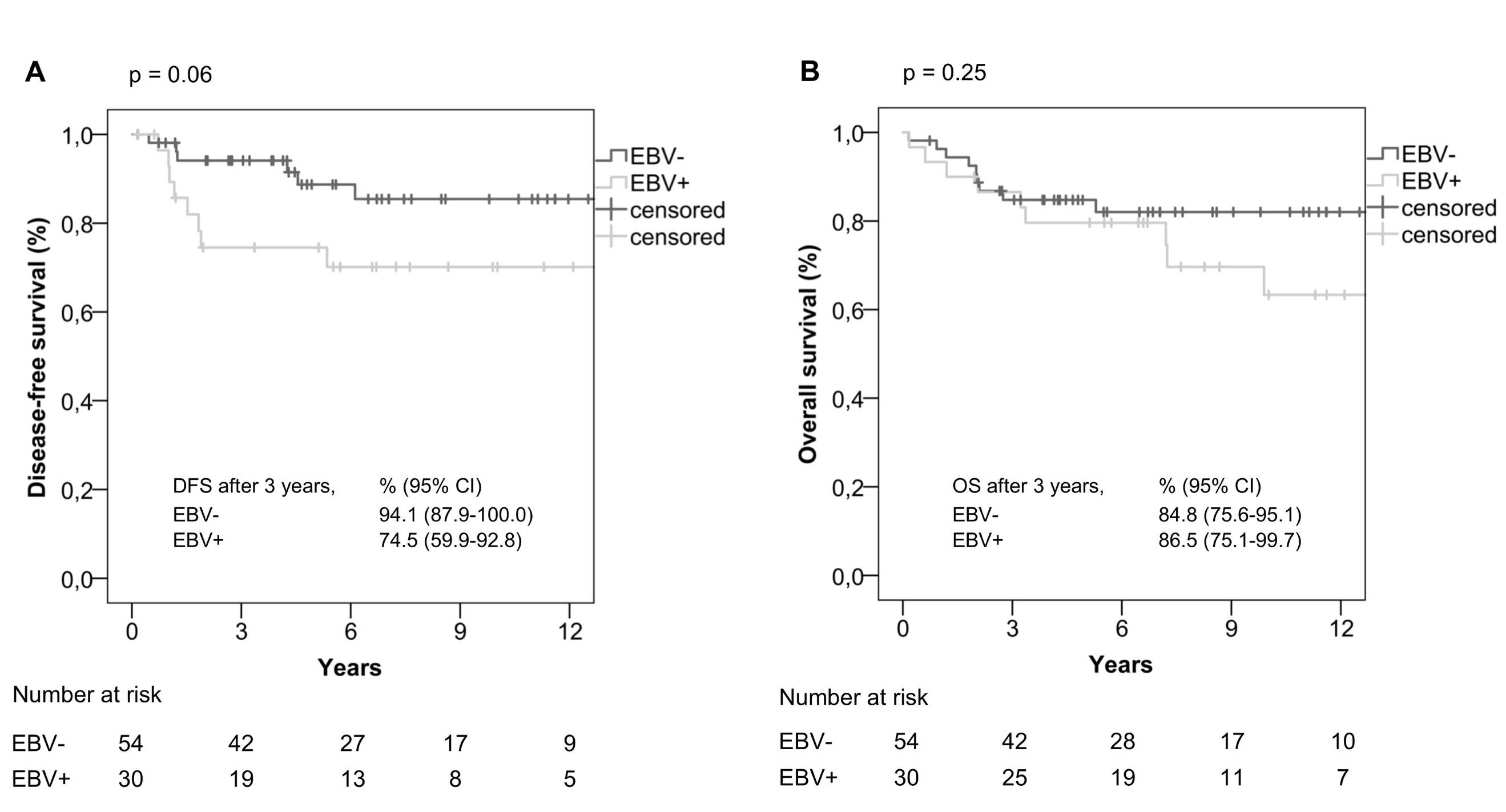

Supplement: Supplementary file 1 — Supplementary information. [file 41598_2020_66010_MOESM1_ESM.docx]
